# Factors associated with longer hospital admission in elderly patients with major neurocognitive disorder

**DOI:** 10.4102/sajpsychiatry.v29i0.2078

**Published:** 2023-10-23

**Authors:** Tarina Steenkamp, Paslius S. Mazibuko, Carla Kotzé

**Affiliations:** 1 Weskoppies Psychiatric Hospital, Institution of Gauteng Health, Pretoria, South Africa; 2 Department of Psychiatry, Faculty of Health Sciences, University of Pretoria, Pretoria, South Africa

**Keywords:** admission, dementia, elderly, geriatric, inpatient, neurocognitive disorder, placement, social support

## Abstract

**Background:**

Major neurocognitive disorder presents many challenges to patients, families and healthcare systems, especially when a patient requires admission to a psychiatric hospital.

**Aim:**

To identify characteristics of older patients with major neurocognitive disorder at risk of prolonged admission in a psychiatric hospital.

**Setting:**

A tertiary psychiatric hospital in Gauteng province, South Africa.

**Methods:**

The authors conducted a retrospective review of the hospital database and clinical files. Clinical and demographic data were collected from the files of 50 inpatients, 60 years and older, who were diagnosed with major neurocognitive disorder and admitted between 2015 and 2019. Anonymised data from patient records were captured on an electronic spreadsheet and analysed using *T*-tests and analysis of variance (ANOVA) to investigate the relationship between patient characteristics and length of hospital admission.

**Results:**

The mean duration of admission was 18.29 months. Involuntary admission status (*β* = 0.239, *p* = 0.049), level of assistance required (moderate level of assistance [*β* = 0.378, *p* = 0.005]; high level of assistance [*β* = 0.336, *p* = 0.015]), availability of social support (*β* = −0.319, *p* = 0.016) and the presence of behavioural or psychological problems (*β* = 0.437, *p* = 0.002) were significantly correlated with longer admission. Using a stepwise regression model, the only significant variable associated with a shorter length of stay was the presence of social support (*β* = −0.512, *p* = 0.009). Age, type of major neurocognitive disorder and number of comorbidities were not correlated with the duration of admission (*p* > 0.005).

**Conclusion and contribution:**

Social support plays an important role in the management of patients with major neurocognitive disorder. The findings in this study highlight healthcare shortages and a need for adequate placement facilities in South Africa for patients who have no other form of support.

## Introduction

Psychogeriatrics is a specialised field of psychiatry that focuses on the multidisciplinary assessment, diagnosis and treatment of elderly patients with psychiatric illness.^1^ As of mid-2020, South Africa’s population was estimated at 59 620 000 people, of whom 5 430 000 were aged 60 years and older. The aged population in South Africa is also growing, showing an average growth of 3% between 2019 and 2020 compared to 1.1% reported over the period 2002–2003.^2^ In South Africa, the increasing population growth, along with the 0.39% increase in life expectancy, which currently stands at 64.12 years,^3^ will lead to an increased burden of disease and costs to the healthcare system.

The South African Health Review report of 2019 predicts that ageing will increase expected health expenditure by 7.9% from 2002 to 2022.^4^ This report found that elderly people are 7.6 times more likely to develop multiple chronic conditions, with 80% of the elderly relying on public healthcare.^4^ Advanced age is a well-known risk factor for developing multiple chronic illnesses, which include psychiatric pathology.^5^ Stein et al.^6^ reported that the elderly (> 65 years) population have a 27.9% lifetime prevalence of developing one psychiatric disorder, 9.6% chance of developing two or more disorders and 2.8% chance of developing three or more disorders in their lifetime.

The occurrence of mental illness in the elderly also has implications for families and healthcare facilities. In Iraq, Ibrahim et al.^7^ reported that the prevalence of mental illness among older patients was 38.7%, with rates in nursing homes more than double than the rates of those who were cared for by their families. The complexity of mental illness likely results in many elderly patients staying in healthcare facilities for extended periods of time.

Long-stay patients have been defined differently across studies. Barnett et al.^8^ considered long length of stay for any patient who was admitted for longer than 28 days. Another case-control study, conducted in London-based acute psychiatric wards, considered prolonged admission to be longer than 6 months.^9^ In Japan, elderly patients comprised an eighth of all admissions to the psychiatric unit, with an average length of stay of 364 days.^10^ They attributed long admissions to the poor health of elderly patients, with many of them having comorbidities requiring additional treatment.^10^ Gigantesco et al.^11^ reported that behavioural disturbance, more specifically the presence of violent behaviour, made their study population four times more likely to require longer admission, in their study on psychiatric patients with prolonged admission in both public and private acute inpatient facilities in Italy.

In South Africa, Aartsma, Groenewald, Koen, Potocnik and Niehaus^12^ examined the patient profile of inpatient admissions to the psychogeriatric unit at Stikland Hospital. They found that most inpatients older than 60 years of age had either a primary or comorbid diagnosis of a cognitive disorder.^12^ The prevalence of physical comorbidities was also found to be high at 74.5%.^12^ Hypertension and hypercholesterolemia were found to be the most common medical comorbidities, with a prevalence of 47.4% and 20.7%, respectively.^12^ Diabetes mellitus was the third most common comorbidity at 14.6%, followed by vitamin B12 deficiency (9.8%), chronic obstructive pulmonary disease (7.1%) and hypothyroidism (5.9%).^12^ The magnitude of challenges that elderly patients face when it comes to identifying their healthcare and long-term needs is underplayed due to the scarcity of local research.^13^

In South Africa, many placement facilities for elderly psychiatric patients require patients to have a certain level of functionality and financial position. These requirements result in restricted access to healthcare because almost half of the South African population live in areas with minimal or no access to adequate health services and care facilities. A Cape Town-based study found that 79% of patients attending a local memory clinic were cared for by family members.^13^ These family members often have to leave their jobs to meet the care needs of patients with a neurocognitive disorder.^13^

A report compiled by strengthening responses to dementia in developing countries (STRiDE) such as South Africa highlighted the challenges faced by elderly people requiring long-term, facility-based dementia care.^14^ Most of these patients were cared for by unpaid, female family members at home.^14^ The public sector facilities that provide long-term care to these patients are mostly underfunded and include non-governmental and religious organisations. The demand for these types of facilities also far outweighs the number of beds that are available. Although there are facilities providing dementia care in the private sector, these facilities are not accessible to most of these patients due to financial restrictions.^14^

Few studies in South Africa have investigated the factors affecting long hospital stays among elderly patients with major neurocognitive disorder. In South Africa, the burden of neurocognitive disorders is expected to increase.^13^ According to the World Health Organization, approximately 58% of the 50 million people globally living with dementia (also known as major neurocognitive disorder) reside in low- and middle-income countries. This figure is expected to triple by the year 2050, with the proportion of people living with dementia in developing countries increasing to 68%.^15^ It is thus important to understand the factors associated with prolonged admission of elderly people living with neurocognitive disorders. In this retrospective study of hospital databases and clinical records, the authors identified the risk factors associated with longer hospital stay among elderly patients with neurocognitive disorder. Understanding these risk factors in the South African setting could allow for the early identification of risk factors in patients requiring longer admission. Early identification of elderly patients at high risk of prolonged hospital admission can help to ensure that adequate arrangements regarding future care are made timeously.

## Method

### Study design

The study followed a descriptive, cross-sectional study design. A retrospective record review of patients admitted to Weskoppies Psychiatric Hospital between 01 January 2015 and 31 December 2019 was conducted.

### Study setting

Weskoppies Psychiatric Hospital is a tertiary-level psychiatric hospital which is in Tshwane, Gauteng, South Africa. The hospital has 854 beds, of which 337 are allocated for acute adult and geriatric admissions and 170 for long-stay adult and geriatric patients. The hospital currently serves approximately 300 patients over the age of 60 years on both an inpatient and outpatient basis.

### Assessment of neurocognitive disorder

Major neurocognitive disorder diagnosis was based on the criteria as set out in the Diagnostic and Statistical Manual of Mental Disorders, Fifth Edition (DSM-5).^16^

### Study population

The authors included the records of all patients aged 60 years or older and patients who turned 60 while being admitted to Weskoppies Psychiatric Hospital, with a major neurocognitive disorder. Files of patients younger than 60 years while admitted as well as missing files or files with incomplete data were excluded from the study.

### Data extraction

Patient files were identified using the hospital’s electronic database. Patients’ age at the time of admission was recorded, primary diagnosis as well as secondary diagnoses, including comorbidities was also recorded. The authors assessed patient files for the presence or absence of behavioural and psychological disturbances including aggression, apathy, depressive symptoms, manic symptoms, agitation, psychotic symptoms and disturbance in vegetative symptoms. The authors also recorded the outcome of each patient’s admission as discharged, death of the patient or ongoing admission. The authors also noted whether the patient was discharged to family or a care facility and whether the patient had any form of support available outside of the hospital context. Support was assessed as the presence of any supportive figure who is willing to accommodate the patient or offer continuous involvement in the care of the patient following their discharge from the hospital.

The admission status of patients was recorded according to the *Mental Health Care Act 17 of 2002*.^17^ Admission status was classified as voluntary, assisted or involuntary status, depending on their capacity to consent to admission and treatment, as well as their willingness to receive care, treatment and rehabilitation on an inpatient basis. The duration of admission in months was calculated from the date of admission until discharge. Patients who were still admitted at the end date of the study had their duration of admission calculated from date of admission until 30 June 2020. The authors defined ‘longer admission’ as admission exceeding 6 months.

The authors also assessed the level of assistance required for activities of daily living (ADLs). Patients who were able to do ADLs independently or required minimal assistance such as reminders were assessed as needing minimal assistance. Moderate and high levels of assistance referred to patients needing continuous or complete supervision or physical assistance, respectively, in performing ADLs.

### Statistical analyses

Anonymised data from 50 patients were captured electronically. Data were analysed using Statistical Product and Service Solutions (SPSS) Data Analysis Software version 27. Categorical variables, including primary and secondary diagnoses, comorbidities, level of assistance required, admission status and the presence of Behavioural and psychological symptoms of dementia (BPSD), were summarised using descriptive statistics in the form of frequencies and proportions. The two continuous variables, age and duration of admission, were summarised using descriptive statistics such as the mean, median and standard deviation (s.d.). *T*-tests and analysis of variance (ANOVA) were used to measure whether there is a significant relationship between the patients’ characteristics and their length of stay in hospital. There was noted to be one outlier in the data set (with a duration of admission of 88 months). Based on this, the test of skewness is statistically significant, with a skewness value of 1.394. After the removal of the outlier, the skewness is still statistically significant, with a skewness value of 0.827. The authors used a multiple regression to assess the relationship between predictor variables and length of hospital stay.

## Results

Data from 50 records were collected. One record was an outlier, with a duration of admission of 88 months, and the data were excluded to prevent skewing of results. Thus, data from 49 participants were analysed.

Regarding aetiology of major neurocognitive disorder, 49% of patients (*n* = 24) were diagnosed with major neurocognitive disorder due to vascular pathology and 51% (*n* = 25) had a diagnosis of major neurocognitive disorder due to other aetiologies. Approximately two-thirds of patients (*n* = 31, 63%) had a secondary diagnosis of psychotic disorder (*n* = 17, 35%), mood disorder (*n* = 11, 22%) or substance use disorder (*n* = 3, 6%), and 37% (*n* = 18) had a diagnosis of a cognitive disorder only.

The number of comorbid conditions ranged between 1 and 3 (Table 1), with the mean number of comorbid conditions being 1.61 (s.d. = 0.885).

**TABLE 1:** Comorbid physical conditions of patients.

Comorbid conditions	*n*	%
Cardiovascular (including hypertension and CVA)	41	84
Dyslipidaemia	14	29
Diabetes mellitus type 2	3	6
HIV	3	6
Substance use	7	14
Epilepsy	6	12

CVA, cerebrovascular accident; HIV, human immunodeficiency virus.

When assessing admission status, 59% (*n* = 29) of patients were admitted as involuntary mental healthcare users and 41% (*n* = 20) as assisted mental healthcare users. Regarding behavioural and psychological disturbances, 51% (*n* = 25) of patients presented with behavioural and psychological disturbances that were troubling to staff throughout their admission period. During admission, 18% (*n* = 9) of patients required a high level of assistance, whereas 37% (*n* = 18) required moderate assistance.

When assessing social support, 51% of patients (*n* = 25) had some form of support available outside of the hospital and 49% (*n* = 24) had no form of support. By the end of the study period, 39% of the patients (*n* = 19) were still admitted to the hospital, 55% (*n* = 27) were discharged and 6% (*n* = 3) were deceased. Of the 27 patients who were discharged, 21 (43%) patients returned to their families and 6 (12%) were sent to a suitable placement facility. The mean age at discharge or end of the study was 68.69 years (s.d. = 5.602, range 60–88 years).

### Multiple regression

The mean duration of admission was 18.29 months (s.d. = 15.933, range 1–52 months).

Behavioural and psychological symptoms were found to be an outlier and removed before using a stepwise regression model. In the model, presence of behavioural and psychological symptoms was coded as 1 and the absence thereof coded as 0. The regression model was statistically significant (*R*^2^ = 0.191, *F*(1, 47) = 11.102, *p* = 0.002) and the presence of behavioural and psychological symptoms was a significant predictor of longer length of stay (*β* = 0.437, *p* = 0.002).

A Pearson correlation coefficient was done to assess the relationship between duration of admission and type of major neurocognitive disorder, number of comorbidities, presence of behavioural and psychological symptoms, level of assistance required, age at discharge or end of study, number of comorbidities, admission status and availability of support. Only three of the independent variables showed significant correlation with the duration of admission. There was a significant positive correlation between the duration of admission and a moderate level of assistance required (*r* = 0.331, *n* = 50, *p* = 0.009), admission status (*r* = 0.290, *N* = 50, *p* = 0.021) and support available (*r* = −0.524, *N* = 50, *p* = 0.000).

Initial regressions, analysing all independent variables of 50 data sets, found that the combination of independent variables explained a total of 24.1% of the variance in duration of admission when adjusting for the number of predictors. The model was not significant (*F*(10, 15) = 1.795, *p* = 0.670) and as could be expected, none of the individual predictors were significant (*p* > 0.05).

Further regressions were done with the three variables showing significant correlation with duration of admission (support, level of assistance required, involuntary admission status) and one outlier record was removed (admission duration of 88 months). In the regression model, support was coded as 1 and no support coded as 0. The combination of independent variables explained a total of 65.6% of the variance in duration of admission (adjusted *R*^2^ = 0.430). The model was significant (*F*(4, 44) = 8.312, *p* ≤ 0.001) and all individual predictors were significant (*p* < 0.05).

The regression model suggests that lack of support (*β* = −0.319, *p* = 0.016), moderate level of assistance required (*β* = 0.378, *p* = 0.005), high level of assistance required (*β* = 0.336, *p* = 0.015) and involuntary admission status (*β* = 0.239, *p* = 0.049) were all predictive of longer admission.

Compared to a low level of assistance, a moderate level of assistance required 12.355 more months in hospital (*p* = 0.005). Compared to a low level of assistance, high level of assistance required 13.703 more months in hospital (*p* = 0.015). Involuntary admission led to an increase of 7.669 months when compared to assisted admission (*p* = 0.049). When support was available, hospital stay tended to be 10.432 months shorter than when this was not the case (*p* = 0.016)

Stepwise regression was used to determine if the availability of support only predicted the duration of admission. The regression model was statistically significant (adjusted *R*^2^ = 0.23, *F*(1, 23) = 8186, *p* = 0.009) and support was a significant predictor of length of stay (*β* = −0.512, *p* = 0.009), suggesting that the presence of support predicted a shorter length of stay. Age, type of major neurocognitive disorder and number of comorbidities were not predictive of the length of stay (*p* > 0.005).

## Discussion

In this study, the authors investigated the factors associated with prolonged admission of elderly patients with neurocognitive disorders in a psychiatric hospital. Secondary diagnoses and comorbidities were common in this group of patients. Behavioural and psychological symptoms were also prevalent. Social support played an important role in determining duration of admission, with patients who had social support less likely to be admitted for lengthy periods.

Many studies have identified barriers to discharging psychiatric patients back into the community. The prominent characteristics associated with prolonged admission to psychiatric facilities include poor social or community support, advanced age and the presence of physical comorbidities.^8,11,12,18,19,20,21,22^ The average duration of admission of patients diagnosed with major neurocognitive disorder in this study was approximately half of that reported in a psychiatric facility in Japan.^10^ The patients in this study spent an average of 18.29 months admitted to hospital. This is markedly longer than the average length of stay recorded in the psychogeriatric unit of Stikland Hospital, which was 53 days.^12^ This may be explained by the study done at Stikland Hospital including minor and major psychiatric pathologies, whereas the sample in this study only included patients with a major neurocognitive disorder. Patients with a major neurocognitive disorder often have a longer length of stay due to the complexity of the pathology.

Most of the patients in this study were diagnosed with a vascular major neurocognitive disorder, with two-thirds also having a comorbid psychiatric diagnosis. Similarly, Kunik et al.^23^ also reported a high prevalence of psychiatric comorbidities, although having a comorbid psychiatric disorder contributed to longer duration of inpatient care in their study. In this study, 59% of the patients were initially admitted as involuntary mental healthcare users, which is in keeping with international findings (60.8%).^24^ Aartsma et al.^12^ reported that 97.1% of patients admitted to the psychogeriatric unit at Stikland Hospital were admitted involuntarily.^12^ Involuntary status was found to be a significant contributing factor to longer admission in this study and in studies from Australia^24^ and the United States.^25^ Patients who require involuntary admission may have more severe symptoms of illness and need to be admitted for longer.

Medical comorbidities may also play a role in the duration of admission. Schubert et al.^26^ reported that patients with dementia had on average 2.4 medical comorbidities, while the patients in this study had an average of 1.61 comorbidities. The most prevalent comorbidities in the analysis in this study included hypertension, dyslipidaemia and diabetes, which is similar to those reported at Stikland Hospital.^12^ This may also explain the high prevalence of vascular-associated major neurocognitive disorder reported in this study. While there is a direct relationship between comorbidities and caregiver burden,^27^ we did not find a direct association between the number of comorbidities and the duration of admission.

In this study, the presence of BPSD and the level of assistance required both influenced the duration of stay.^28^ These factors may be intertwined. In this study, almost half of patients presented with BPSD that were troubling to staff, which is not unusual.^29^ Gigantesco et al.^11^ reported that behavioural and functional impairment contributes significantly to length of stay.^11^ Patients who display BPSD are often unmanageable outside of a structured hospital environment, which may explain the need for longer admission in patients who present with these symptoms. These patients may also require a high level of assistance with ADL. Families of the patients in the setting of this study are not always equipped with the resources to adequately care for patients who need assistance; thus, these patients may need to remain hospitalised or require long-term care in a care facility setting. Unfortunately, longer hospital stay may lead to further functional decline.^30^

In this study, the main association with longer admission was a lack of social support. Patients with major neurocognitive disorder can place high burden on caregivers due to various factors. Caregivers may have a limited understanding of the illness and poor coping skills, which are exacerbated by patient-related factors including behavioural problems and poor functional status. Caring for older persons with disabilities may have a substantial impact on the financial position of families. Family members often had to leave their work to care for the impaired family member, as well as spend more on health services, which results in lower income.^31^ This is not feasible for most families in the current economic climate in South Africa. South African primary care settings are also not able to provide intensive support programmes for families and patients.^32^ The need for prolonged admission can also be explained by the lack of suitable placement facilities. Only 12% of patients in this study were sent to a placement facility. Patients may not qualify for admission to long-term care facilities due to their physical and psychological requirements or behavioural difficulties. There are also few suitable facilities available to care for patients with no other form of financial support. Although there are approximately 1150 residential facilities for older persons in South Africa, only 415 facilities are registered with the Department of Social Development. Of these, only eight are fully subsidised and run by the government. In contrast, there are more than 1000 private residential facilities and retirement villages country-wide where residents and families are responsible for the full expense.^33^

The lack of services and support for older people with major neurocognitive disorder raises concerns about stigmatisation and infringements on their human rights. Budget allocations within the health system have to be reconsidered to include major neurocognitive disorder as a priority condition to promote the well-being and sense of dignity of patients living with dementia.^34,35^

## Study limitations and recommendations

This study had several limitations including difficulty in obtaining information from files due to information only being available in a paper-based system. Files had to be excluded from analysis due to missing or incomplete data. The small sample size of this study at a single hospital makes it difficult to generalise the results to other settings. The diagnoses captured in the file was based on DSM-5 criteria and were accepted as accurate in the setting of a tertiary academic hospital with strict quality control measures.

Areas of future research could include investigating the effect of different symptoms of BPSD on length of admission and cross-cultural studies to examine the role of social support in hospitalisation outcomes across different cultures or countries. Examining the economic implications of longer hospital admission in older patients with dementia could identify cost-effective strategies to improve social support and reduce admission periods. It is recommended that frameworks should be developed to address the lack of support and training for caregivers and to guide policies that will address the need for person-centred care for these patients.

## Conclusion

Social support plays an important role in the management of patients with major neurocognitive disorder. There are few resources available to meet the needs of patients who have no other form of support. Thus, there is a great need for caregiver training, community-based care and adequate placement facilities to assist with the care and support of these patients. Focus should also be placed on primary care and prevention. Families should be supported from the initial contact with their primary mental healthcare provider to help better educate and equip them with the skills needed to provide long-term care to their family members with a major neurocognitive disorder.

## Contribution

Major neurocognitive disorder is an illness that places a lot of strain, not only on the health sector but also on the families and patients themselves. Research on and services for older patients with the diagnosis of a major neurocognitive disorder is scarce in South African settings. This study has assisted in identifying clinical characteristics that put these psychiatric patients at risk for longer admission, as well as protective factors. It highlights the urgent need for interventions and policies to address the insufficient services for this vulnerable population to promote and protect their human rights.
